# A Theoretical Framework on the Determinants of Food Purchasing Behavior of the Elderly: A Bibliometric Review with Scientific Mapping in Web of Science

**DOI:** 10.3390/foods10030688

**Published:** 2021-03-23

**Authors:** Khaled Alhammadi, Luna Santos-Roldán, Luis Javier Cabeza-Ramírez

**Affiliations:** Department of Statistics, Econometrics, Operations Research, Business Organization and Apllied Economics, Faculty of Law, Business and Economic Sciences, University of Cordoba, 14071 Cordoba, Spain; ep2alalk@uco.es (K.A.); r62caral@uco.es (L.J.C.-R.)

**Keywords:** elderly, older consumer, purchasing behavior, buying behavior, good purchases, consumer behavior

## Abstract

The past few years have seen significant demographic changes in most regions, including an increased elderly population. Subsequently, elderly citizens comprise an important market segment of consumers, with the food industry one of the most affected areas in this context. However, food market managers previously believed that elderly consumers’ needs were stereotyped in nature. The lack of focus on this sector, therefore, left elderly consumers as an untapped market, without realizing the financial independence of this segment regarding their nutrition. This research will attempt to provide the key determinant factors on elderly consumers’ behavior related to food. For that purpose, a complete literature review of more than 123 papers regarding these concepts has been carried out. Once analyzed, we highlight the common insights to give clear guidance for supermarket managers and food manufacturers to have a better knowledge of the reasons behind elderly people’s food acquisitions.

## 1. Introduction

Age is one of the most critical factors that influences or affects consumer behavior [1] because it determines the way of life of an individual. For elderly consumers, the age-related changes drive their specific choices of products and services, which tend to be different from younger adults, and, therefore, have major significance in consumer behavior and marketing [2]. As Drolet et al. [3] stated in their research, the influence of aging on consumers and their shopping experiences and purchasing decision-making is generally complicated, since it involves various behaviors and mental processes for different aging individuals. In this regard, based on the literature, some of the mental processes related to age that play a key role in influencing consumer behavior include factors of cognition, sensory functioning, motivation, and affect. Therefore, it is of great importance for marketers to use different approaches to identify the specific needs and demands of the aging population to achieve positive consumer behavior.

It is understandable that marketers cannot expect people of all ages, whether they are 20 or 70 years, to have the same desires and needs for products and services. In fact, people’s habits evolve with age and time, resulting in changing needs for products and services. This is why age is one of the key factors marketers must look at when considering consumer behavior analysis [4,5,6]. The present work aims to advance the understanding of the research hitherto carried out on the purchasing and consumption habits of the elderly. To meet this objective, we prepared a synthesis and a systematic review of the literature [7], based on science maps [8,9], in order to highlight the aspects identified by authors who have studied this theme. Additionally, we explore the main research topics hitherto discussed, and propose a comprehensive theoretical framework based on previous literature. This research continues the path started among others by Yoon et al. [10], Host et al. [11], and Zniva and Weitzl [12] in an effort to group and consolidate the accumulated knowledge.

### 1.1. Elderly Consumers and Their Importance

When elderly consumers are discussed, it is important to note that over the lifespan of an individual, changes tend to occur in respect of their choices and actions. Without addressing the needs of this particular segment of consumers, it can be said that it represents a loss for businesses, as they miss out on targeting a significant percentage of the population as customers [13].

In addition, one of the key factors that make elderly consumers an important sector to focus on is that there has been a significant change in demographics over time. It is known that thanks to improved overall living standards and health conditions of people, along with greater advances in healthcare systems, longevity has increased and older people live longer. This, therefore, suggests that this particular consumer segment (elderly consumers) is continuously growing, and hence becomes a more important segment to address by marketers [14,15,16].

The importance of elderly consumers also significantly lies with their changing food habits and patterns, which in turn influence their purchasing decisions. The food consumption habits of consumers hold major significance among marketers, supermarkets, and retailers, as the sale of such products has a major influence and impact on businesses. Hence, if food habits change with age, it becomes important to highlight such changes in elderly consumers so that marketers can accordingly offer them their preferred products [17,18]. In this regard, the factor of heterogeneity holds another major significance, in that elderly consumers are important for marketers and retailers to consider, analyze and target as a separate consumer group [12,19]. Consumer behavior needs to be studied from the first moment the consumer is faced with a series of decisions to be made. These vary according to the type of product or purchase situation which defines their behavior. Elderly consumers have to face a wide variety of decisions when selecting a product in the market and they depend on their available resources (time, money, and knowledge).

### 1.2. Challenges of Elderly and Food Purchase

The current COVID-19 pandemic has not only caused global concerns on health issues of individuals, it has also changed the behavior of consumers, mainly because going out and shopping normally is now considered a threat to the lives of people due to the threat of the virus. Examples of changed behaviors include stocking up of essential items at home, especially in times of lockdown when going out is prohibited, owing to the need for social distancing. Unlike previous times, new products have been added to the shopping list as essential items, such as sanitizers, masks, gloves, and other protective gear, for virus protection purposes [20].

This situation represents a particular challenge for older people. Because of lockdowns and layoffs in companies to manage business losses, older people are mostly being affected, losing their jobs, experiencing major disruptions in their normal service activities, and losing their retirement savings. On the other hand, their health vulnerabilities are on the rise, along with other associated issues such as depression and anxiety driven by suffering from the disease or being in a state of panic and threat [21]. Furthermore, as they are facing challenges with their earning and savings, it also has an impact on how much they can spend on their purchases.

Thus, there is an overall change in the traditional ways and habits of shopping styles and purchases. Online purchases have increased drastically during the COVID-19 pandemic t, which reflects how technology is taking over. Customers are increasingly considering online shopping options instead of physically visiting the stores [22]. Due to the technological advances and increased involvement of information and communications technology, ICT is driving the advanced designs of supermarkets and their services, and elderly consumers are experiencing more difficulties shopping in the supermarkets. Additionally, the use of ICT, lack of proper signage, and lack of effective customer support is further reducing the motivation levels of the elderly consumers to consider supermarkets for shopping. Although they are trying to learn quickly and use technology to avoid visiting stores, they do not feel comfortable with the Internet and online shopping features. On the other hand, there is the possibility that the new habits that people are adopting could become habitual habits in the future, which could mean that older consumers will gradually adopt new experiences and shopping options [23].

## 2. Materials and Methods

In accordance with the objectives proposed in this work, two methodological approaches were combined. First, we used the PRISMA protocol [7], a proven procedure in the field of systematic literature reviews and meta-analyses that provided transparency and replicability to the review [24]. Thus, we gathered a representative set of documents related to the behavior of buying and choosing food in older people. Next, we applied scientific mapping techniques through co-word analysis [25,26], which allowed us to approach the intellectual structure of previously selected documents [27,28]. Both techniques have been used in previous work on the elderly [29,30].

### 2.1. Systematic Review of the Literature

**Figure 1:**
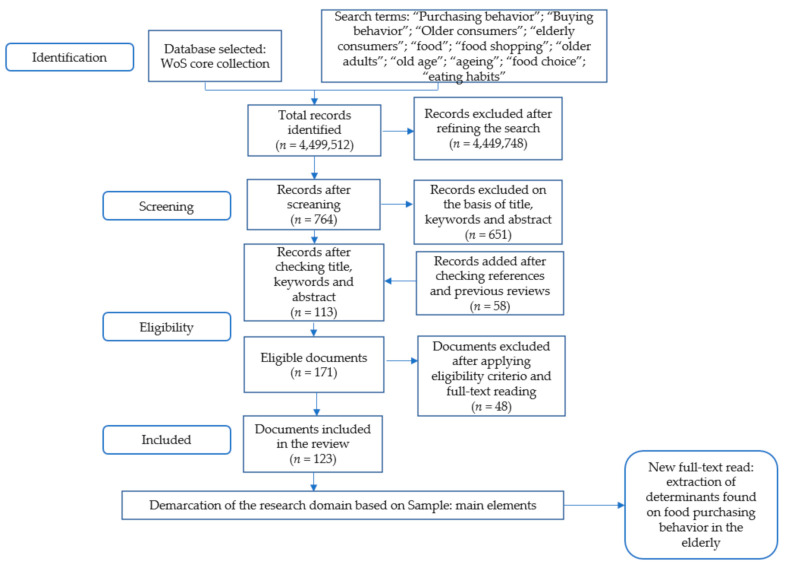
Flow diagram adapted according to the PRISMA protocol.

The PRISMA protocol integrates a series of successive stages that allow organizing the selection criteria used and reaching the relevant knowledge in a given field of study [7]. Figure 1 summarizes the whole procedure:

The identification included the choice of the information source. To this effect, we opted for the use of the Web of Science (WoS) Core Collection, since it ensured that unique patterns of document classification according to research areas were used; in addition, it had a sufficient volume of documents, scientific quality, and debugging [31]. The search terms were selected in line with previous research in the field of behavioral studies in the elderly [10,11,12]. The temporal coverage was not limited, nor the documentary typology, which allowed the inclusion of articles from conferences and other sources such as books or book chapters that may be of interest in the analysis of such a specific research domain. The initial search was screened and refined using the WoS “Marked list” function, applying multiple combinations with the selected keywords and the “Refine Results” option. In the initial searches, all records that could not be discarded without reading the full text were kept, and then the references contained in the selected studies were screened and compared with documents included in previous reviews [10,11,12].

There were three eligibility criteria. In the first place, the theme had to directly address consumer behavior or choice in older people. Second, the document had to address food consumption, even if it was part of a larger investigation or at least indirectly. Thirdly, it must be possible to retrieve the full text. For that purpose, a first reading was carried out, discarding those documents without a specific theme or methodology or those that did not provide research data, objectives, or results clearly.

The document search process began in November 2020 and was updated until February 2021, adding new records as a result of new publications. The second reading of documents was carried out and the determinants found in the sample were synthesized, from which a summary was extracted with the objectives and variables reported in the 123 documents constituting the review.

### 2.2. Systematic Map: Co-Word Analysis

The so-called science maps are spatial representations that help to visualize the relations that arise between documents, in other words, they show links between authors, bibliographic references, journals, disciplines, and predominately words [25,26,27]. In relation to the research objective, a network analysis was proposed that delved into the content of the documents through the co-occurrence of keywords. This methodology is especially suitable if the intention is to advance the intellectual structure of a specific part of a domain or research field [26,28]. As pointed out by Choi et al. [32], the keywords indexed in documents and those contained in titles and abstracts are essential for the identification of significant topics within a specific research area.

The visual representation of this type of map is usually done with bibliometric software [19]. In this research, we used VOSviewer [9] and SciMAT [8]. The first tool was used to offer a broad vision of the research domain focused on the determinants of food buying behavior in older people and, the second, to illustrate the “motor”, “basic”, “emerging”, “developed”, or “isolated” themes within the domain [33]. The combination of both instruments increased the advantages and offered new possibilities for global interpretation [34,35,36].

VOSviewer stands out for its graphic power; it is positioned as a particularly suitable instrument [37] to show the complete domain of research. With this tool, the centrality of a word (node) determines its relative position in the network. The software calculates the centrality and strength of all the words; the greater the weight is, the larger the node or word size is. Links between nodes represent the number of times words appear together, and the strength of the link is illustrated by its thickness. For its part, Scimat incorporates more options related to the scientific mapping workflow [35,37]; for example, it is easier to identify the documents that are generating specific thematic networks and are highlighted within the set. Table 1 shows the data related to the configuration used with the two tools. In VOSviewer, a minimum frequency of occurrences of 2 was determined, since it was intended to broadly show the largest possible number of thematic networks. A thesaurus file was used to debug and group synonymous terms or the singular and plural of certain words (for example: aged, age; behaviors, behavior, etc.). In the case of Scimat, we proceeded in the same way, using its grouping and debugging tools.

On the other hand, Scimat represents the themes (keywords) in two-dimensional diagrams where the “*x*” axis shows the centrality and the “*y*” axis shows the density of the thematic groupings. Centrality establishes the degree of interaction of a topic or word with others, in other words, its importance for the development of the domain. Density reflects the internal strength of the subject with respect to others, that is, the ability to maintain and develop over time. As a result, a diagram composed of four quadrants was generated: (a) Motor themes, with high centrality and density; (b) Basic themes, with high centrality and low density; (c) Emerging or declining themes, with low centrality and density, and (d) Developed and isolated themes, with low centrality and high density. For the creation of the networks, the algorithm of the simple centers and the equivalence index were applied. More details on the configuration and application of the software can be found in [8,27,33].

## 3. Results

The sample consisted of a total of 123 documents [4,5,6,10,13,14,15,16,17,18,19,38,39,40,41,42,43,44,45,46,47,48,49,50,51,52,53,54,55,56,57,58,59,60,61,62,63,64,65,66,67,68,69,70,71,72,73,74,75,76,77,78,79,80,81,82,83,84,85,86,87,88,89,90,91,92,93,94,95,96,97,98,99,100,101,102,103,104,105,106,107,108,109,110,111,112,113,114,115,116,117,118,119,120,121,122,123,124,125,126,127,128,129,130,131,132,133,134,135,136,137,138,139,140,141,142,143,144,145,146,147,148,149] whose analysis period included works published and indexed in the database in the interval between 1973 [125] and February 2021.

### 3.1. Description of the Sample Documents

**Figure 2:**
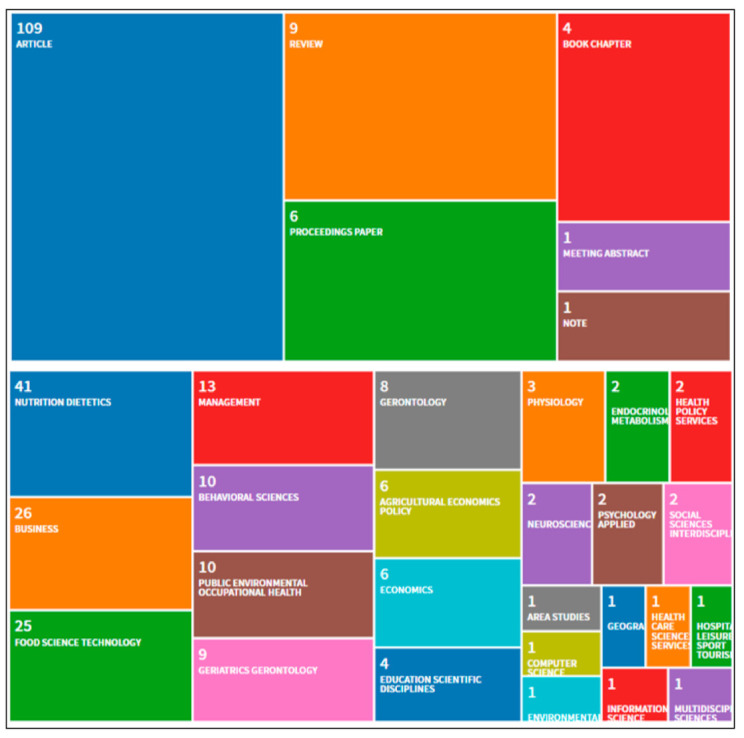
Documentary typology and predominant indexing categories.

The first documents dealt with the mobility and transportation difficulties faced by the elderly to satisfy basic food needs [125], their satisfactory and unsatisfactory experiences with purchased products [99], and attitudes and preferences regarding purchased foods [118]. On this basis, progress was made towards greater complexity in the topics and variables used to obtain a better understanding of the results. For example, the most recent documents addressed the influence of the senses on eating behavior [6], food insecurity, loneliness, and social support among the elderly [44], or the consumer’s assessment of specific foods [4]. Figure 2 shows the document typology and the indexing categories in WoS, taking into account that the same document can be indexed in several categories or typologies simultaneously.

The 123 documents mainly included articles, quantitative and qualitative works (obtained from samples of different sizes, local, regional and national, of different age groups, usually from 50 years), descriptive statistics and rigorous tests, segmentations [138], and general reviews [16,74,148]. As expected, the main indexing categories were aligned with the main theme of this review, such that documents cataloged as nutrition and dietetics [64], business [10] and food, science and technology [89] stood out.

### 3.2. Global Visualization of Accumulated Research Using VoSviewer

**Figure 3:**
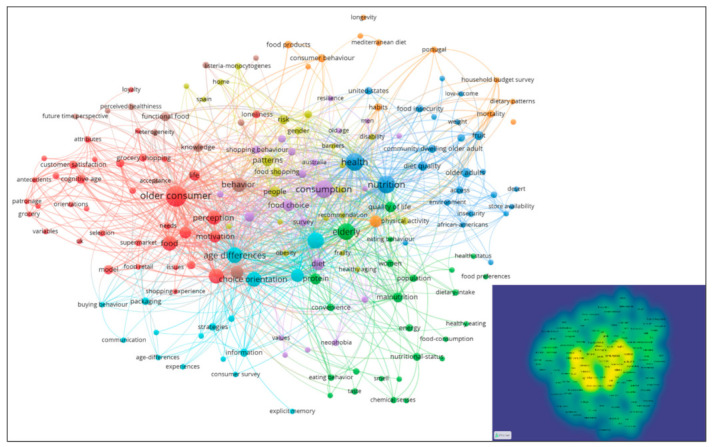
Map of thematic clusters detected and density visualization.

The keyword network with the criteria established in the methodology section with a minimum of two occurrences was composed of 170 words. The most representative keywords generated different clusters. Those with the strongest links and number of occurrences designated the grouping by assigning a label. Table 2 shows the summary with the main groupings and Figure 3 shows their visualization, as well as the level of saturation.

The first thematic grouping was characterized by grouping themes related to the motivation of the elderly consumer [79,95,106,108], and the different segments into which these themes could be grouped [84,138,140]. The second grouping included papers that addressed characteristic issues such as quality of life or the nutritional status of older people [19,69]. The third cluster included topics related to the health of the individual and the quality of the diet [50,120]. The fourth grouping mainly addressed patterns of behavior in the purchase of food [14,68,72,126], as well as risks [116] or diseases associated with their eating [41,106,137]. The fifth grouping delved into generational differences related to consumption [111,146,147]. The sixth cluster stood out for including the different ways of understanding consumption orientation according to the age groups found in the elderly [75,95,97,123], as well as other influential variables such as the available information [53,90,127]. The seventh grouping was very varied, it included topics aligned with the different determinants that could be found to understand the multiple patterns of consumption [43,48,55,65,68,80,112,122,136]. Finally, the last grouping included topics related to additional variables such as attitudes [103,118], knowledge [16,139], or heterogeneity [17,138,140].

As a whole, the research domain represented through the sample of documents did not reflect signs of saturation (Figure 3, bottom); however, it included a multitude of overlapping topics that became intertwined and progressively hindered a specific understanding.

### 3.3. Conceptual Field Evolution Using Scimat

The documents indexed a total of 678 terms or keywords that were reduced to 459 keywords by grouping singular and plural, or synonymous terms, as indicated in the methodology section. After executing the Scimat scientific mapping software with the established configuration (Table 1), 14 strategic topics were obtained related to the research domain on food purchases and consumption habits in elderly people. Table 3 lists the centrality and density of each of the clusters, as well as the main documents linked to each cluster. The topics with greater centrality and density were configured as engines of the research domain. Among them were “Countries”, “Antecedents”, “Meat-consumption”, “Population”, “Age-Differences”, and “People”. As basic and cross-cutting themes, “Determinants” and “Senior-Marketing” appeared. Thirdly, four emerging or declining themes emerged: “Accessibility”, “Loneliness”, “Food-Products”, and “Aging-population” and, finally, two rapidly developing or isolated themes: “Supermarket” and “Odor”.

**Figure 4:**
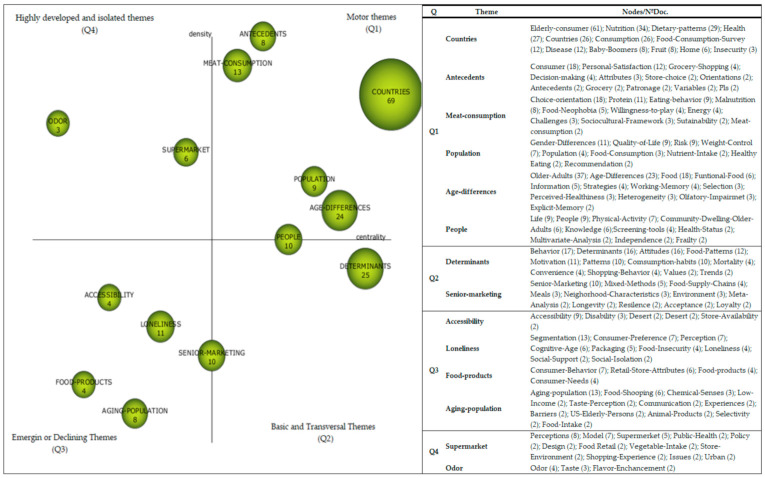
Matrix diagram depicting the performance of the research themes by the number of documents.

Due to the fact that some of the labels assigned to the different groupings could be too generic, their interpretation was extended to the set of the most representative terms contained in each one of them, as well as to the number of documents in which they appeared, as shown in the (Figure 4).

The motor themes located in the first quadrant (Q1) were mainly composed of groupings that included attributes and characteristics that were best adapted to the elderly that were shown to be decisive in understanding purchasing and consumption behavior [10,11,12,138]. It was fundamentally about a person’s individual elements, including their habits and customs, as well as the analyses carried out in different countries and geographical contexts [56,57,108]. Among the most prominent were common topics such as “Nutrition” [41,90,148], “Health” [41,93,112], “Disease” [41,106,137], “Personal-Satisfaction” [46,92,99], “Orientations” [88], “Gender Differences” [49,110], “Quality of Life” [67,69,120], and “Age differences” [53]. These groups made substantial contributions to the development of the domain since its inception and were still in full force.

The basic and cross-cutting topics were positioned in the second quadrant (Q2) and corresponded to two main topics—“Determinants” [48,55,65,68,80,112,122,136] and “Senior Marketing” [82,107,132]. Among the most prominent groups were those focused on the determinants of consumption and eating patterns [14,72,126], which included purchasing habits and behaviors and actions carried out in the field of marketing specifically aimed at the different segments that made up the group of elderly people. These themes extended indirectly or crosswise through the rest of the groupings detected (Q1, Q3, Q4), complementing the motor themes of the domain, and were fundamental in providing a better understanding of the rest of the groupings generated.

The emerging or declining topics located in the third quadrant (Q3) dealt mainly with attributes linked to establishments [81], types of stores [38], retail stores [39,111], availability [104,124], choice [40,108], environment [17,94] or source of satisfaction and dissatisfaction in the elderly clients [46,92,99].

Finally, quadrant four collected highly developed or isolated themes and mainly integrated groupings related to the senses [62,89,122,129] and department stores [17,18,63,104,109,115].

### 3.4. A Integrative Theoretical Framework through Thematic Analysis

**Figure 5:**
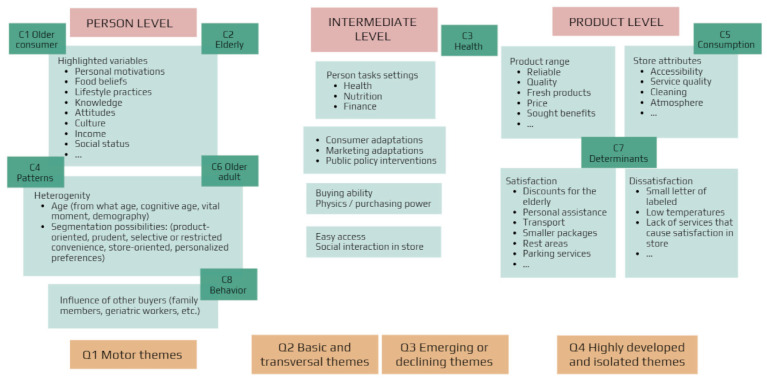
Theoretical framework and positioning of thematic clusters obtained with *VoSviewer* and *Scimat.*

Once the topics through which the set of representative documents of the research domain were configured, a review of the literature and a full-text reading of the set were carried out. Subsequently, a multilevel perspective was applied [150] based on the progress reported in previous reviews [10,11,12]. In this way, three levels were established. The first level, called the person level, collected the individual characteristics of the elderly person, including their individual traits, age, generation or cohort, goals, motivations, and general psychography [10,138]. The second level, called the intermediate level, was based on the person-task adjustments, which linked the characteristics with the changes that the individual had undergone relative to the passage of time; for example, changes in health, nutrition, or finances. The third level, called product, linked the food product with the characteristics and attributes of the establishments where purchases were made and which were a source of satisfaction and dissatisfaction. Finally, the thematic groupings obtained with VoSviewer and Scimat were positioned according to the degree of proximity to each of the three levels, as shown in (Figure 5).

## 4. Discussion and Conclusions

Based on the theoretical model proposed in this article and the results provided by its analysis, three different levels are shown:

First of all, the “personal level” was widely considered. To explain the behavior of the elderly consumer, we select the factors that can be considered personal and that constitute fundamental variables [1,77,92,106,121]. To begin with, the age and stage of the individual’s life cycle influence the changes present in the structure of products and services. On the other hand, due to the growth of the elderly population and the adaptation of markets to their needs, aspects such as profession, level of education, and purchasing power are also included here, mainly [51,52,123]. However, age stands out as the main study variable. The concept of third age is commonly used to refer to those over 65 years old; however, there are studies that differ and mark the figure by more than 55 or even 75 years old. In this sense, the World Health Organization considers people aged 65 years or more as older people and people aged 85 years or over as very old people. Ultimately, the consensus opinion in the literature refers to the fact that the group of elderly people is not a homogeneous group and requires other more sophisticated segmentation factors.

On the other hand, both emotion and motivation are representative aspects of the non-cognitive part of human thought, which are defined by their intrinsic relation with factors associated with the practice of consumption [10]. The factor of affection could be reviewed as a more determining factor in the behavior among elderly consumers. In this regard, it could be deduced that their emotions further contribute to their personality and the perceptions which drive their preference for particular brands, products, or services and, hence, determine their choices of offering as well as shopping preferences [77,122]. Furthermore, cultural factors can be highlighted, understanding culture as a group of beliefs, rules, values, knowledge, attitudes, and habits established over time and shared by individuals from the same community. Other classifications corresponding to cultural factors are the subculture and social class, the latter being the common characteristics shared by individuals of the same social status.

Likewise, within this level, social factors are of paramount importance. The social groups of reference through a feeling of union, common rules, and objectives affect consumer behavior in the process of formalizing opinions [44,105]. The family stands out especially, as the modality with the greatest influence on the consumer due to its durability superior to the rest. Consequently, on a large number of occasions, the family is the driving force behind the purchase [14,110].

From this level, it can be inferred that, in recent years, as a consequence of an aging world population, the attention paid to elderly consumers has increased in the scientific literature. Nowadays it is recognized that changes in the number of working-age and elderly people influence consumption and savings patterns. Undoubtedly, older customers play and will play an important role in retail spending in a convulsed economic future in the aftermath of the global pandemic.

Secondly, an “intermediate level” in which the works of authors that affect the convenience of adaptation to this market segment are observed. It is true that for retailers it becomes crucial to firstly address and understand the specific needs and expectations of elderly consumers, considering them a completely different marketing segment to target [42,55,83,117]. However, the question nonetheless arises as to whether they really understand the elderly consumers. As could be reviewed, usually with aging, they experience a systematic decline in cognitive processing that includes memory issues and deficient executive functioning of the brain. Age effects also become visible in the speed of their information processing during mental operations skills [70,75,141].

In this regard, it could be considered that the emotions of elderly consumers further contribute to their personality and perceptions, which drive their affection for particular brands, products, or services and, hence, determine their choices of offering as well as shopping preferences [10,66,108]. Moreover, it could also be perceived that if a brand is able to develop suitable promotional measures such as advertising capable of initiating positive emotions in consumers, positive purchase actions can also be expected from the elderly consumers, since in their case, it is based on the affection factor rather than on cognition [79]. This means that marketers could review the role of advertising and marketing in influencing the affection of older consumers, as it affects the behavior of this type of consumer.

The benefits related to elderly consumers, however, lie in the scope which marketers have in developing new products to address the specific needs that this segment has, which are different from those of other customer segments. This implies that businesses can focus on innovations and newer developments in products to address this segment, as well as increasing profits from sales if they can rightly fulfill the needs and requirements of elderly consumers [116]. In relation to the necessary adaptation, we can indicate a deficit of studies focused on the role of elderly consumers in digital environments. When it comes to technological challenges, elderly consumers face difficulties in adapting to technological advances, including their physical challenges or lack of comfort with the use of technology because they lack confidence in their skills and abilities [132]. The elderly consumers need more assistance, which in turn becomes a barrier for them. However, at the same time, a major positive factor has been found associated with the use of technological advances by senior customer groups. Although they initially tend to have greater challenges with understanding technology and making use of it, once they are able to embrace technology and its benefits, the elderly people engage more with Internet-based activities, which provides major benefits for both the elderly consumers and the businesses marketing products to them [30]. This opens up new scope and opportunities for businesses to connect with this particular segment, understand their needs and hence offer them specific products and services. The benefits are also for elderly consumers, as they would then not lag behind other segments in respect of having information on various products and services, and hence, will make better purchasing decision-making.

Following this, it is highly recommended to managers from supermarkets to provide guides for using online systems, remove financial barriers, and prioritize a conversational user interface with audio, as well as developing apps focused on elderly users that are intuitive, with a clear layout, bigger letters, and customized guidance.

Thirdly and last, a “product level” is warned. A significant research gap could be realized in determining the purchase response of elderly consumers to supermarkets and the impacts of promotions of supermarkets on this market segment, considering the available sources of previous research findings. As could be obtained from the reviews, the choice of and responses to supermarkets have a significant association of people’s choices of diets and lifestyles, which they look for being fulfilled with offerings made by the supermarkets [18,133,145]. The sales promotions of supermarkets are largely based on the displays of products on the shelves in multiple aisles and customers tend to get influenced by the information they have from the product packaging [67,82,115]. The shape and size of the products, the information shared on the packaging, and the packaging materials play a crucial part in promoting the products to the customers, which in turn determines their behavior [130]. Older people face difficulties in shopping from the high and low shelves where different products are arranged in a supermarket. The height of the shelves, signs and displays, size and proportions, and labeling are not effectively suitable for elderly consumers in most cases, particularly when they have long been comfortable with traditional grocery store style shopping. At the same time, there are changing consumption patterns among the elderly consumers, which, however, seem to be less addressed by the supermarkets in their marketing and promotions [17,63].

In aspects to consider, we highlight the different physical needs in relation to the service and design of supermarkets, the predilection or rejection of department stores, or the desire for personal treatment by shop assistants [81,113]. Deficiencies and failures in these factors are the cause of elderly consumers abandoning a brand and losing loyalty. As den Uijl, Jager, de Graaf, Waddell, and Kremer [60] stated in their research paper, the loyalty of elderly consumers is more associated with their affection rather than their cognition. In this regard, it could be realized that affection, cognition, and customer satisfaction are the three key factors determining the loyalty of elderly consumers. When affection is considered, it represents the emotional and mental ways in which an individual interprets information, the actuation of their perception, which in turn determines their positive or negative feelings and affections for other people or objects.

In parallel, the location of the supermarket is a very important aspect, especially when it is a point to be evaluated by elderly consumers [17,108]. Numerous studies have concluded that both the distribution of products in the supermarket and the equipment for their transport are two of the most evaluated points [52]. Due to the physical deterioration of buyers, comfort is the main deciding factor. Within this convenience, the most relevant components are: easy entry and exit points, informed and trained sales staff, or celerity at checkout counters. From what has been mentioned, the physical environment/surroundings in supermarkets, malls, or restaurants are critical factors that can determine the level of influence on the minds of elderly customers towards driving them to a purchase. The effectiveness, comfort, and presentation of physical surroundings also determine how and why customers would choose one marketing brand over another, which is particularly true when it comes to physical shopping food experiences for customers. In order to achieve sales, therefore, managers need to focus on this factor significantly, ensuring that they offer a comfortable and safe physical surrounding to their elderly customers. Such physical surroundings are also significant in relation to the sales and promotion techniques considered by a marketer. In cases of supermarkets and hypermarkets, while the marketer can offer various discounts and offers on their products, it is also important that the physical surroundings are suitable. Unless the physical surroundings and shopping experience of the consumers are suitable and comfortable, discounts and offers or any promotional activities rarely would have significant impacts on the consumer.

### Limitations and Future Lines of Research

The results presented here, including the theoretical framework developed from the previous literature, are not without limitations nor do they pretend to offer a single vision of reality. Our purpose is to make a small contribution to the structuring of an emerging and now booming research domain. The main limitation of this work stems from the choice of a single source for its development, the Web of Science Core Collection. Being aware that it is a controversial decision, it was made to mitigate possible errors derived from the use of bibliometric software. However, it opens the door to future analyses by combining other sources that help to complete and contrast the findings presented here. Secondly, the interpretation of a scientific map is extremely complex. Despite the detailed reading of all the included papers and the monitoring of a structured process, this study remains descriptive-qualitative and may harbor any bias unintentionally introduced by the researchers involved. Thirdly, only documents in English were reviewed; however, our work opens the door to future research that should be carried out based on the events that have occurred in recent times. The incidence of the period of isolation in the eating habits of the elderly is undoubtedly one of the greatest challenges for the future.

A more detailed analysis of the purchasing behavior of older people by different age ranges, gender, and nationality are proposed as future lines of research. Finally, this paper represents the first step of an on-going project with a variety of actions focused on the elderly. The authors are developing a survey in line with the research model including the constructs of loyalty, physical surroundings, buying behavior, and sales promotion techniques (point of purchase, advertisement, free sample, price discounts, two for one, and refunds). All these results will contribute to a proposed app to ease online shopping for this market segment and help address a very demanding need that is now fundamental to remain in business for managers of supermarkets and distributors in the food industry.

## Figures and Tables

**Table 1:** Software configuration.

VoSviewer V.1.6.16		Scimat V.1.1.04	
Item	Characteristic/Value	Item	Characteristic/Value
Type analysis	Co-occurrence	Select periods	Period (1973–2021)
Unit	All Keywords	Unit	Author’s Words
Counting method	Full counting	Data reduction	Frequency reduction = 2
Normalization Method	Association Strength	Kind of matrix	Co-occurrence
Layout	Attraction = 2/Repulsion = 0	Network reduction	Minimum value = 1
Clustering	Resolution = 1.00/Min Cluster size = 10	Normalization Method	Equivalence index
Visualization Scale	Network and overlay = 1.27	Cluster Algorithm	Simple centers (Max network = 12; Min = 3)
Weights	Occurrences	Mapper	core mapper
Labels size variation	Min. Strength = 0/Max. Lines = 500	Quality measures	all
Minimum numbers of occurrences of a keyword	2	Longitudinal map	Equivalence index

**Table 2:** Thematic cluster detected in global research.

Cluster/Color/Label	N° Keywords	First Five Keywords (Links, Total Link Strength, Occurrences)
C1/red/Older Consumer	36	Older consumers (108, 255, 37); Food (76, 139, 17); Perception (73, 122, 15); Segmentation (65, 96, 11); Motivation (57,91,11)
C2/Green/Elderly	25	Elderly (94,188, 23); Protein (56, 87, 10) Quality of life (59, 84, 9); Malnutrition (42, 70, 8); Nutritional Status (32, 42, 5); Energy (26, 29, 4)
C3/Blue/Health	24	Health (105, 229, 27); Nutrition (103, 227, 26); Diet Quality (40, 56, 7), Older adults (37, 44, 7); Community Dwelling older adults (40, 55, 5)
C4/Yellow/Patterns	22	Patterns (57, 81, 10); People (52, 84, 9); Risk (39, 58, 7) Food Shopping (32, 37, 6); Aged Related Diseases (33, 39, 4)
C5/Purple/Consumption	21	Consumption (88, 185, 26); Food Choice (40, 70, 10); Diet (63, 103, 12); Baby Boomers (39, 58, 7); Survey (32, 46, 5)
C6/LightBlue/Older adult	17	Older adult (89,166, 21); Age Differences (82, 147, 20); Choice Orientation (75, 132, 18); Aging (58, 83, 13); Information (25, 35, 5)
C7/Orange/Determinants	13	Determinants (57, 101, 11); Consumer Behavior (27, 33, 5); Food Products (18, 23, 4); Mortality (26, 46, 4); Mediterranean diet (18, 19, 3)
C8/Brown/Behavior	12	Behavior (62, 11, 16); Attitudes (58, 103, 13); Knowledge (42, 54, 6); Functional Food (34, 53, 6); Heterogeneity (21, 27, 3)

**Table 3:** Thematic cluster and core documents detected by *Scimat*.

Themes	Q	Centrality	Centrality Range	Density	Density Range	Core Documents (Highly Cited)
Countries	1	279.74	1	41.19	0.86	[4,5,14,15,16,18,38,39,41,42,45,47,48,50,51,52,55,56,57,58,59,60,62,65,68,69,72,73,74,75,77,78,79,80,81,83,84,85,91,92,93,97,100,104,105,106,109,111,112,113,114,115,116,119,120,121,122,123,130,132,134,137,138,139,142,143,146,147,149]
Antecedents	1	132.68	0.64	73.88	1	[13,17,18,39,40,96,108,133]
Meat-consumption	1	131.61	0.57	59.02	0.93	[4,19,41,43,48,60,65,70,77,103,112,139,140]
Population	1	148.47	0.79	27.26	0.64	[19,41,49,52,55,56,105,112,147]
Age-differences	1	164.8	0.86	23.14	0.57	[10,13,39,46,50,61,62,66,69,71,77,87,88,107,108,110,111,116,127,129,130,139,140,141]
People	1	140.05	0.71	21.99	0.5	[19,46,49,67,77,84,93,134,137]
Determinants	2	167.87	0.93	19.1	0.43	[5,14,19,43,47,51,59,60,65,79,91,103,106,107,108,113,115,119,120,130,135,138,146,147,149]
Senior-marketing	2	131.34	0.5	15.13	0.21	[16,75,85,92,104,107,116,117,123,139]
Accessibility	3	56.75	0.21	18.29	0.36	[81,104,105,124]
Loneliness	3	124.73	0.36	18.02	0.29	[5,17,44,55,56,58,61,67,108,114,138]
Food-products	3	44.11	0.14	13.91	0.14	[57,101,133,149]
Aging-population	3	107.29	0.29	13.89	0.07	[6,13,62,110,121,132,134,143]
Supermarket	4	130.51	0.43	32.02	0.71	[17,18,63,104,109,115]
Odor	4	31.83	0.07	33.33	0.79	[89,122,129]

## Data Availability

The sample of papers and all data are available upon request to any of the authors.
